# Mechanical Reinforcement of Ethylene Vinyl Acetate (EVA) Nanocomposites Prepared from Masterbatch of Cellulose Nanofibers Wrapped with Ethylene Vinyl Alcohol (EVOH)

**DOI:** 10.3390/polym18020167

**Published:** 2026-01-08

**Authors:** Hyungrai Kim, Hyewon Lee, Seokkyoo Seo, Heejung Jang, Jeyoung Park

**Affiliations:** 1 Department of Chemical and Biomolecular Engineering, Sogang University, Seoul 04107, Republic of Korea; enxyme3328@sogang.ac.kr (H.K.); hwlee@sogang.ac.kr (H.L.); 2 Hanwha TotalEnergies Petrochemical Research Institute, Seosan 31900, Republic of Korea

**Keywords:** cellulose nanofibers, ethylene–vinyl alcohol, ethylene–vinyl acetate, masterbatch processing, interfacial compatibility, mechanical properties

## Abstract

Ethylene–vinyl acetate (EVA) copolymers are widely used in packaging, films, foams, and adhesives because of their softness and optical clarity; however, their relatively low mechanical strength limits broader applications. In this study, a scalable masterbatch strategy was developed to reinforce EVA by introducing TEMPO-oxidized cellulose nanofibers (T-CNFs), pre-encapsulated within an ethylene–vinyl alcohol (EVOH) matrix. EVOH acted as a compatibilizer, establishing robust hydrogen bonding with T-CNFs (evidenced by a 2.73-fold increase in the hydrogen bonding index) and thereby promoting their uniform dispersion and strong interfacial adhesion in the hydrophobic EVA phase. The resulting nanocomposites demonstrated significant improvements in mechanical performance, achieving a maximum 1.54-fold increase in tensile strength and a 1.42-fold increase in Young’s modulus compared to neat EVA. These findings highlight a practical route to produce bio-based, mechanically enhanced EVA nanocomposites with potential for industrial-scale applications.

## 1. Introduction

Ethylene–vinyl acetate (EVA) copolymers are widely employed in packaging, footwear, adhesives, and photovoltaic encapsulants due to their flexibility, transparency, and processability [1,2,3,4]. Their mechanical and thermal properties can be tuned by adjusting the ethylene/vinyl acetate ratio [5,6,7]; however, the intrinsically low tensile strength and stiffness of EVA restrict its use in applications demanding higher load-bearing capacity.

In parallel, growing interest in sustainable, bio-based reinforcements has driven the development of polymer nanocomposites, where nanoscale fillers enhance strength, modulus, and toughness [8,9,10,11]. Among them, cellulose nanofibers (CNFs) have attracted considerable attention because of their renewable origin, high aspect ratio, and excellent specific mechanical properties, rendering them promising candidates for advanced eco-friendly composites [12,13,14,15]. Yet, the direct incorporation of hydrophilic CNFs into hydrophobic matrices such as EVA typically leads to severe agglomeration, moisture sensitivity issues, and poor interfacial adhesion [16,17,18].

To overcome these incompatibilities, various chemical surface modifications, reactive compatibilization routes, and the use of intermediate-polarity polymers have been explored [19,20,21,22,23,24,25]. Ethylene–vinyl alcohol (EVOH), with its hydrogen-bonding capability and intermediate polarity, has emerged as an effective compatibilizer for CNFs, and masterbatch approaches offer a scalable route toward improved dispersion in hydrophobic matrices [26,27,28,29].

However, many reported strategies rely on additional chemical grafting or complex processing, raising concerns regarding cost, environmental impact, and industrial feasibility [30]. To address these limitations, we strategically selected EVOH as the masterbatch carrier. Its amphiphilic structure—possessing both ethylene segments compatible with EVA makes a like-dissolves-like effect and abundant hydroxyl groups for robust hydrogen bonding with T-CNF—allows it to function as an ideal physical bridge between the hydrophobic matrix and hydrophilic filler.

**Figure 1:**
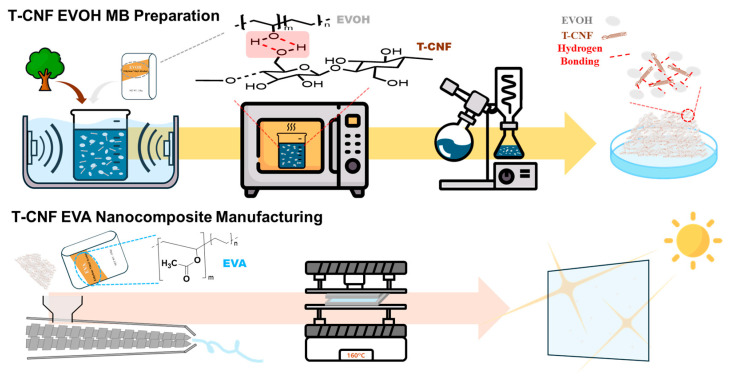
Schematic illustration of the fabrication process of EVA-based nanocomposites using T-CNF/EVOH masterbatch.

Consequently, this study presents a novel, industrially scalable strategy to overcome T-CNF agglomeration in EVA using an EVOH-assisted masterbatch system via melt compounding. The specific objectives of this research are to (1) investigate the morphological dispersion of T-CNFs facilitated by the EVOH; (2) quantify the enhancement in mechanical properties, particularly tensile strength and Young’s modulus; and (3) verify the thermal stability of the masterbatch to ensure its usage for practical industrial applications. Figure 1 suggests a flow diagram of the experimental workflow, allowing for a clear visualization of the sequential steps involved in this study.

## 2. Materials and Methods

### 2.1. Materials

Isopropyl alcohol (IPA) was purchased from Daejung Chemicals & Metals (Siheung, Republic of Korea). T-CNFs were purchased by Anpoly Inc. (Pohang, Korea). Poly(ethylene-co-vinyl alcohol) (EVOH) (VOH = 68 mol%, *M*_w_ = 26,000 g mol^−1^, *T_g_* = 58 °C) and Poly(ethylene-co-vinyl acetate) (EVA) (VA = 28 mol%, *M*_w_ = 50,000 g mol^−1^, *T_g_* = 25 °C) were provided by Hanwha TotalEnergies Petrochemical (Seosan, Republic of Korea). All reagents were used without further purification.

### 2.2. Preparation of EVOH/T-CNF Masterbatch

#### 2.2.1. Evaluation of T-CNF and EVOH Dispersibility

**Figure 2:**
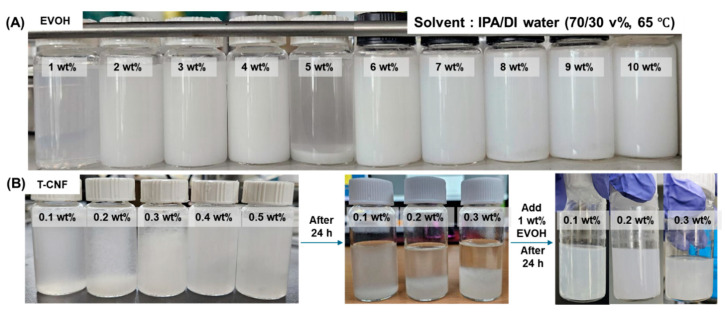
Dispersibility image of (**A**) EVOH and (**B**) T-CNF in 70:30 volume ratio of IPA to DI water at 65 °C.

To establish suitable conditions for homogeneous dispersion of T-CNF and EVOH, a cosolvent system was selected. Based on reported solubility data, a 70:30 (*v*/*v*) mixture of isopropyl alcohol (IPA) and deionized (DI) water, maintained at 65 °C, was chosen as the working solvent system to facilitate EVOH solubilization [31]. T-CNF suspensions were prepared at 0.1–0.5 wt% (0.1 wt% increments), while EVOH solutions were prepared at 1–10 wt% (1 wt% increments). All solutions were ultrasonicated at 65 °C for 24 h and subsequently kept in a 65 °C water bath for an additional 2 h. The dispersion state was then visually evaluated as seen in Figure 2.

#### 2.2.2. Physical Modification and Recovery of T-CNF/EVOH Masterbatch

To prepare the T-CNF/EVOH masterbatch, a 1 wt% EVOH solution in 70:30 (*v*/*v*) IPA/DI water was ultrasonicated at 65 °C for 24 h (frequency: 40 kHz, power: 400 W; Power Sonic 420, Hwashin Technology, Gwangju-si, Gyeonggi-do, Republic of Korea), yielding a transparent and stable dispersion that indicated successful solvation. T-CNF was then incorporated into the EVOH solution at 5, 10, 20, and 40 wt% relative to the EVOH mass. The mixtures were further ultrasonicated at 65 °C for an additional 24 h under the same operational conditions to ensure uniform dispersion and promote interfacial interaction.

To promote hydrogen bonding and initiate partial solvent removal, the resultant dispersions were subjected to microwave irradiation (3 × 15 s, microwave 1000 W, LG Electronics, Seoul, Republic of Korea). The mixtures were subsequently concentrated by rotary evaporation under reduced pressure at 40 °C. The preconditioned masterbatch was fully dried in a vacuum oven at ambient temperature for 72 h, followed by freeze-drying. The obtained solid was pulverized using a shear mixer to yield a uniform powder, which was vacuum-sealed and stored until further use.

For clarity, the samples containing 5 and 40 wt% T-CNF with respect to EVOH were hereafter referred to as MB5T/E and MB40T/E, respectively. These masterbatch formulations were employed in the melt-compounding stage to evaluate their mechanical and thermal reinforcement in EVA composites.

### 2.3. Melt Blending of T-CNF/EVOH Masterbatch with EVA Using Twin-Screw Extrusion

EVA pellets were freeze-dried and ground using a suspension mixer to obtain a particle size comparable to that of the T-CNF/EVOH masterbatch (MB) powder. To minimize moisture uptake, both EVA and MB powders were pre-dried in a vacuum oven at 60 °C for 24 h. The powders were then physically premixed at a weight ratio of 1:99 (MB:EVA) and compounded using a counter-rotating twin-screw extruder (MiniLab, Thermo Fisher Scientific, Waltham, MA, USA).

Extrusion was performed at 160 °C with a screw rotation speed of 100 rpm. A cyclic compounding process was employed for 5 min to ensure homogeneous dispersion of the masterbatch within the EVA matrix. The resulting extrudate strands were pelletized into uniform granules using a mechanical cutter and subsequently sealed in polyethylene bags at room temperature until further characterization.

### 2.4. FTIR-Based Analysis of Hydrogen Bonding Between T-CNF and EVOH in the Masterbatch

Fourier-transform infrared (FTIR) spectroscopy was employed to investigate the functional groups and intermolecular interactions in the masterbatch. Spectra were recorded using an FTIR spectrometer (FTIR-4100, Jasco, Tokyo, Japan) in attenuated total reflectance (ATR) mode. Measurements were performed over the 4000–600 cm^−1^ range with a resolution of 4 cm^−1^, and each spectrum was averaged over 64 scans to enhance the signal-to-noise ratio. The spectra were analyzed to evaluate hydrogen bonding between T-CNF and EVOH as well as to confirm the chemical integrity of the processed materials.

### 2.5. Morphological Analysis

The morphology of the masterbatches and EVA composites was examined using a field-emission scanning electron microscope (JSM-7100F, JEOL, Tokyo, Japan) operated at an accelerating voltage of 5 kV. Samples were cryo-fractured in liquid nitrogen perpendicular to the pressing direction to obtain clean cross-sectional surfaces. The fractured surfaces were sputter-coated with a ~5 nm platinum layer to prevent charging and enhance image quality.

SEM images were acquired at both low and high magnifications to evaluate the dispersion of T-CNF within the EVOH masterbatch and its distribution in the EVA matrix after melt blending. Particular attention was given to interfacial adhesion, fiber embedding, filler aggregation, and void formation in order to assess the microstructural integrity and effectiveness of the masterbatch strategy.

### 2.6. Thermal Analysis

The thermal stability and transitions of the masterbatch and composite systems were evaluated by thermogravimetric analysis (TGA) and differential scanning calorimetry (DSC). All measurements were conducted under a nitrogen atmosphere with a flow rate of 50 mL min^−1^.

For TGA, approximately 10 mg of each sample was placed in a platinum crucible and heated from 40 °C to 700 °C at a rate of 10 °C min^−1^. The onset of thermal degradation was defined as the temperature at 3%, 5%, and 10% weight loss (*T*_d3_, *T*_d5_, *T*_d10_). All measurements were performed in triplicate, and the average values are reported.

DSC was employed to investigate melting and crystallization behaviors. Samples (~5–8 mg) were sealed in aluminum pans and subjected to a three-step heating–cooling–heating program: initial heating from 40 °C to 300 °C at a rate of 10 °C min^−1^, holding isothermally for 10 min, cooling to −40 °C at a rate of 10 °C min^−1^, and reheating to 300 °C at a rate of 10 °C min^−1^. From the resulting thermograms, the cold crystallization temperature (*T*_cc_), melting temperature (*T*_m_), and corresponding enthalpies (Δ*H*_m_) were determined. All DSC measurements were carried out in triplicate to ensure reproducibility.

### 2.7. Mechanical Properties

The mechanical properties of the EVA composites containing T-CNF/EVOH masterbatches were evaluated using a universal testing machine (AGS-X, Shimadzu, Kyoto, Japan) (Type V specimen [32]). Composite films were prepared by hot pressing at 150 °C under a pressure of 1 MPa (≈10 atm) for 15 min to obtain sheets with a thickness of ~0.3 mm. Dog-bone-shaped specimens with dimensions of 26.3 mm × 3.2 mm × 0.4 mm were cut by press cutting machine.

Tensile testing was carried out at a crosshead speed of 100 mm min^−1^ using a 10 kN load cell. Tensile strength and elongation at break were determined at fracture, and Young’s modulus was calculated from the initial linear portion of the stress–strain curve (evaluated at ~0.2% strain). For each composition, five specimens were tested, and the mean values with standard deviations are reported.

## 3. Results

### 3.1. Preparation of Masterbatch and Evaluation of Physical Compatibility

A masterbatch approach was designed to improve the compatibility of T-CNF with hydrophobic EVA by exploiting the amphiphilic properties of EVOH. The strategy relies on hydrogen bonding between the hydrophilic surfaces of T-CNF and the hydroxyl groups of EVOH, thereby encapsulating T-CNF within the EVOH matrix. Consequently, the outer hydrophobic segments of EVOH are expected to enhance interfacial affinity with EVA during melt blending.

The masterbatch was prepared using a 70:30 (*v*/*v*) IPA/DI water mixture as the dispersion medium, which enabled stable solubilization of EVOH and T-CNF. EVOH was first dispersed in the cosolvent at 65 °C for 24 h until a transparent solution was obtained. T-CNF was subsequently added and ultrasonicated for 24 h to promote homogeneous distribution. The resulting dispersion was concentrated by microwave and rotary evaporation and appeared homogeneous, suggesting favorable interaction between T-CNF and EVOH [33].

Figure 2A illustrates the concentration-dependent stability of EVOH in a 70:30 (*v*/*v*) IPA/DI water mixture at 65 °C. EVOH formed a homogeneous solution up to 4 wt%. However, at 5 wt%, the system exhibited sedimentation, likely due to increased intermolecular aggregation [34]. At 6 wt% and above, the dispersion transitioned into a non-fluidic gel state [35]; solvents were trapped by high-density physical crosslinks, resulting in a stable gel. This gelation, driven by extensive hydrogen bonding between EVOH chains, significantly increased the medium’s viscosity but hindered processability.

In contrast, as shown in Figure 2B, the T-CNF suspension (without EVOH) showed limited long-term stability, with visible sedimentation occurring within 24 h at concentrations exceeding 0.2 wt%. Notably, the addition of 1 wt% EVOH to the T-CNF suspension, followed by microwave treatment, successfully restored and maintained a stable, turbid dispersion. This suggests that EVOH chains effectively wrap the T-CNFs, providing steric and hydrogen-bond-mediated stabilization through a robust hydrogen-bonded interface. These results confirm that the EVOH-mediated masterbatch strategy is a feasible approach for ensuring the uniform integration of T-CNFs into the composite matrix.

The superior dispersion stability afforded by EVOH can be attributed to its unique role as a polymeric compatibilizer, distinguishing it from conventional low-molecular-weight surfactants. While traditional surfactants often migrate to the surface or degrade during high-temperature melt blending, the high molecular weight of EVOH ensures that the protective wrapping layer remains intact on the T-CNF surface [36,37]. Moreover, compared to maleic anhydride-grafted compatibilizers, which primarily rely on localized chemical bonding [38], the continuous hydrogen-bonded network formed by EVOH’s vinyl alcohol units provides more uniform encapsulation. This prevents the re-aggregation of nanofibers more effectively, as the hydrophobic ethylene segments of EVOH simultaneously ensure high thermodynamic compatibility with the EVA matrix, a dual-functionality seldom achieved by single-component stabilizing agents.

**Figure 3:**
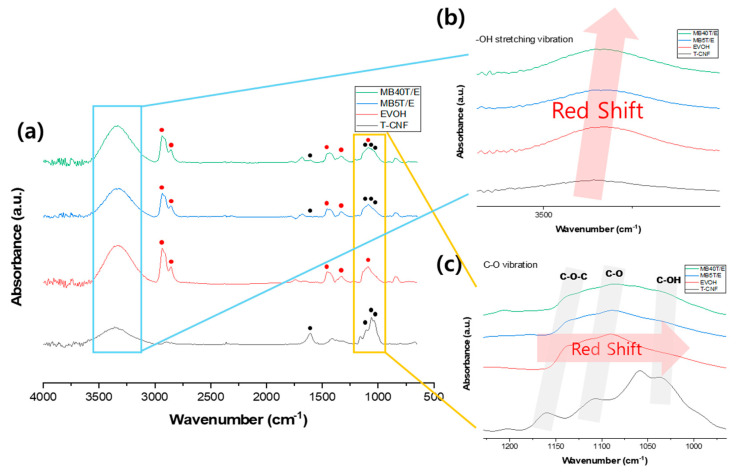
FTIR analysis of –OH stretching and C–O vibrational shifts in T-CNF/EVOH blends. (**a**) FTIR spectra of neat components and masterbatches. (**b**) Enlarged view of red shift in –OH stretching vibration. (**c**) Enlarged view of red shift in C–O vibrational modes.

FTIR analysis was conducted to investigate molecular interactions within the masterbatch. Figure 3 presents the comparative FTIR spectra of the masterbatch, neat EVOH, and T-CNF. Significant peak shifts in –OH stretching (Figure 3b) and C–O vibrations (Figure 3c) highlight the molecular-level interactions achieved through the masterbatch design. As summarized in Table 1, the characteristic absorption peaks of both T-CNF and EVOH were clearly observed in the masterbatch spectra. The –OH stretching vibration, originally at 3360 cm^−1^ in neat T-CNF, shifted to 3329 cm^−1^ in MB5T/E and 3321 cm^−1^ in MB40T/E. The red shift suggests enhanced hydrogen bonding between T-CNF and EVOH, indicative of improved interfacial interactions [39].

To quantitatively validate this interaction, the hydrogen bonding index (HBI) was calculated based on the ratio of the integrated areas of hydrogen-bonded (A_bonded_) to free (A_free_) hydroxyl groups [40,41]. The HBI value increased remarkably from 3.01 for neat EVOH to 6.30 for MB5T/E and further to 8.23 for MB40T/E. This increasing trend confirms that the abundant hydroxyl groups of T-CNF facilitate the formation of a robust hydrogen-bonding network within the EVOH matrix [42,43].

Crucially, the observed red shifts and the substantial increase in HBI correlate directly with the macroscopic mechanical enhancement. The stronger hydrogen-bonded interface indicated by these shifts facilitates more efficient load transfer from the EVA matrix to the T-CNF reinforcements. By restricting the molecular mobility of the polymer chains at the interface and ensuring robust interfacial adhesion, this molecular-level interaction leads to the significant improvements in tensile strength and Young’s modulus observed in the mechanical testing.

Similarly, the C–O–C asymmetric stretching band of neat EVOH, located at 1160 cm^−1^ [44], shifted to 1136 cm^−1^ (MB5T/E) and 1137 cm^−1^ (MB40T/E), while the C–O stretching vibration of T-CNF at 1108 cm^−1^ shifted to 1090 cm^−1^ (MB5T/E) and 1087 cm^−1^ (MB40T/E). Notably, the magnitude of the shifts in the C–O–C and C–O bands (approximately 21–24 cm^−1^) is remarkably higher than the values typically reported for nanocellulose-reinforced composites using conventional compatibilizers [45,46,47]. This pronounced red shift indicates a stronger intermolecular interaction, suggesting that the EVOH chains not only physically coat the T-CNFs but also form an integrated interfacial layer with a high degree of molecular-level coupling. Such a robust interface is instrumental in ensuring that the mechanical stiffness of the T-CNFs is effectively translated to the composite bulk [48,49].

### 3.2. Morphological Analysis of T-CNF/EVOH Masterbatches and EVA Composites

**Figure 4:**
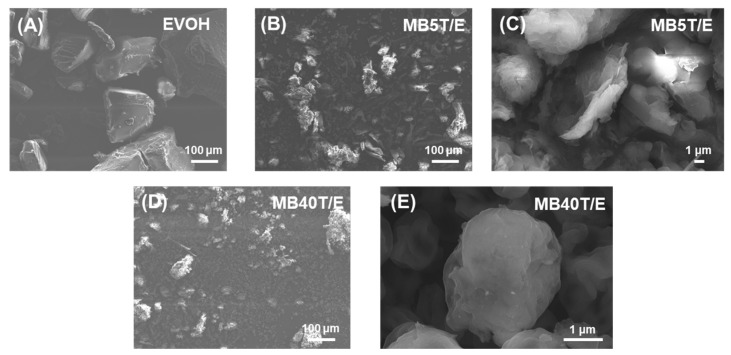
SEM images of (**A**) neat EVOH, (**B**) MB5T/E at magnifications of 150× and (**C**) 5000×, respectively, and (**D**) MB40T/E at magnifications of 150× and (**E**) 20,000×, respectively.

**Figure 5:**
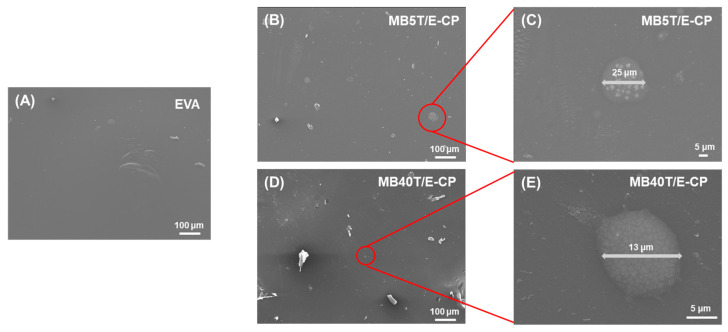
SEM images of (**A**) neat EVA, (**B**) MB5T/E-CP at magnifications of 150× and (**C**) 1000×, respectively, and (**D**) MB40T/E-CP at magnifications of 150× and (**E**) 4000×, respectively.

The morphological characteristics of neat components, T-CNF/EVOH masterbatches (MB), and their corresponding EVA composites were analyzed via scanning electron microscopy (SEM) (Figure 4 and Figure 5).

As shown in Figure 4, the morphology of the masterbatches was significantly influenced by the T-CNF loading ratio. Neat EVOH (Figure 4A) exhibited a relatively smooth and homogeneous surface, whereas neat T-CNF displayed an entangled fibrous network with a rough texture, typical of nanocellulose structures [50,51]. In MB5T/E (Figure 4B,C), the excessive amount of EVOH relative to T-CNF led to incomplete hydrogen bonding between the two components, resulting in the irregular aggregation of EVOH and the formation of relatively large, non-uniform particles. In contrast, MB40T/E (Figure 4D,E) exhibited a distinct morphological shift; the nanofibers and EVOH formed well-defined, spherical micro-particles with a significantly reduced diameter of approximately 3–5 μm. This suggests that a higher T-CNF loading facilitates a more balanced interaction with EVOH, promoting the formation of finer hydrogen bonding masterbatch domains [52].

The dispersion state within the final EVA composites is presented in Figure 5 and the final loading levels of T-CNF in the EVA composites, compared using different master batches, are summarized in Table 2. When melt-blended with EVA, the masterbatches retained their discrete domain characteristics while being distributed throughout the matrix. Consistent with the initial MB particle size, MB5T/E-CP (Figure 5B) showed localized clusters as large as 25 μm, reflecting the coarse nature of the low-concentration masterbatch. However, MB40T/E-CP (Figure 5D) displayed much finer localized domains, with an average size of approximately 13 μm.

While minor localized entanglements were observed at high concentrations, the significant reduction in the size of these domains in MB40T/E-CP compared to MB5T/E-CP confirms a more effective dispersion. Furthermore, no distinct voids or delamination were observed at the interface between the encapsulated domains and the EVA matrix, indicating robust interfacial adhesion. This refined dispersion, driven by the smaller initial MB particle size in the 40 wt% formulation, is considered a key factor in the enhanced mechanical reinforcement of the EVA composites.

To further investigate the reinforcing efficiency and interfacial strength, the cross-sectional fracture surfaces of the tensile-tested specimens were examined (Supplementary Figure S1). In the composite where T-CNF was directly incorporated into the EVA matrix without encapsulation, the fracture surface exhibited clear signs of structural failure originating from the T-CNF domains, characterized by irregular tearing and fiber pull-out. This suggests poor compatibility and stress concentration at the raw fiber–matrix interface.

In contrast, the EVA composites reinforced with the EVOH-wrapped T-CNF masterbatches displayed a remarkably clean and stable fracture morphology. The absence of noticeable debonding or voids at the fracture site indicates that the EVOH encapsulation layer acted as an effective interfacial bridge, facilitating smooth stress transfer from the EVA matrix to the T-CNF. This superior surface stability in the masterbatch-based composites, as compared to the direct mixing approach, provides definitive evidence that the EVOH encapsulation strategy significantly enhances interfacial adhesion and structural integrity in the final composite film.

### 3.3. Thermal Stability Enhancement Through T-CNF/EVOH Masterbatch Integration

#### 3.3.1. Improved Thermal Stability of T-CNF/EVOH Masterbatches via Hydrogen Bonding

**Figure 6:**
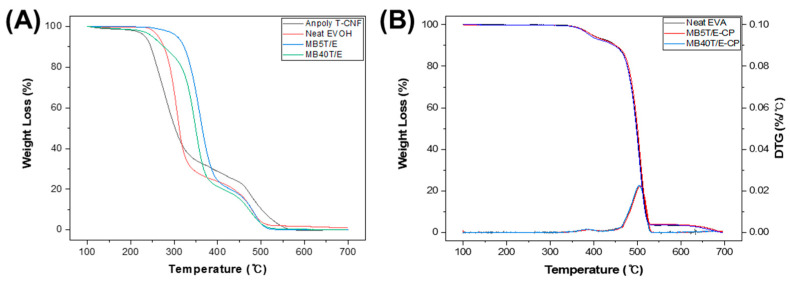
TGA curves of (**A**) neat T-CNF and T-CNF/EVOH masterbatches and (**B**) neat EVA and T-CNF/EVOH MB/EVA composites.

The thermal stability of the T-CNF/EVOH masterbatches was investigated using thermogravimetric analysis (TGA) to explain the effects of hydrogen bonding and nanocellulose encapsulation within the EVOH matrix [53,54]. The decomposition temperature at 5% weight loss (*T*_d5_) was selected as the primary parameter to evaluate thermal stability, and the corresponding results are summarized in Table 3 and Figure 6. The choice of *T*_d5_ as the degradation criterion is intended to exclude the minor weight loss resulting from the evaporation of absorbed moisture or residual processing solvents, which typically occurs below 150 °C. By focusing on *T*_d5_, we can more accurately assess the inherent thermal resistance of the EVOH-wrapped T-CNF network and its influence on the EVA matrix under conditions that mimic industrial melt-processing.

The *T*_d5_ of MB5T/E was determined to be 307 °C, representing a significant increase of 74 °C compared to neat T-CNF (*T*_d5_ = 233 °C). This remarkable enhancement is primarily attributed to the formation of a dense, percolated network stabilized by robust hydrogen bonding between the T-CNF and EVOH. Unlike cellulose nanocrystals (CNCs), which typically possess lower aspect ratios and often exhibit discrete dispersion, the high-aspect-ratio T-CNFs in this study facilitate the construction of a long-range 3D network. This structure functions as a physical barrier that creates a tortuous path, effectively hindering the diffusion of volatile degradation products and restricting the segmental mobility of the EVA matrix.

By contrast, MB40T/E exhibited a *T*_d5_ of 250 °C. While this still reflects an improvement over neat T-CNF, the effect was less pronounced than that of MB5T/E. Interestingly, SEM observations (Figure 4) revealed that MB40T/E displayed a finer physical distribution and more well-defined spherical morphology than MB5T/E, suggesting that the stoichiometric balance at this ratio promotes ideal particle formation.

However, this structural refinement does not directly translate to superior thermal resistance due to a starvation effect of the EVOH wrapping layer. At 40 wt% loading, although the T-CNFs are successfully organized into compact spheres, the available EVOH is spread too thin to fully insulate the vast surface area of the nanofibers. This results in a lower density of interfacial hydrogen bonds and a thinner protective barrier compared to the EVOH-rich MB5T/E. Consequently, while MB40T/E achieves better physical dispersion, the diminished thickness of the encapsulation layer allows for earlier thermal degradation. This indicates that the interfacial quality and the thickness of the protective coating, rather than mere physical shape or dispersion, are the more critical determinants of thermal stability [44,53].

#### 3.3.2. Thermal Stability of EVA Composites Reinforced with T-CNF/EVOH Masterbatches

The thermal stability of the EVA composites was quantitatively evaluated through TGA (Table 4 and Figure 6B). While T-CNF is inherently susceptible to thermal degradation (with *T*_d5_ of approximately 233 °C), its incorporation into the EVA matrix through the masterbatch strategy resulted in remarkably well-preserved thermal stability. Specifically, the *T*_d5_ of MB5T/E-CP and MB40T/E-CP decreased by only 0.6% (2 °C) and 1.8% (6 °C), respectively, compared to neat EVA (*T*_d5_ = 338 °C). Similarly, the *T*_d5_ values showed minimal reductions of 0.2% (1 °C) for MB5T/E-CP and 1.5% (7 °C) for MB40T/E-CP.

The reasoning behind this minimal thermal penalty, despite the addition of thermally sensitive nanofillers, lies in the synergistic wrapping effect of EVOH. Quantitatively, the fact that the degradation onset shifted by less than 2% indicates that the EVOH layer forms a robust thermal barrier, effectively shielding the T-CNF from the high-temperature environment during the CP process. Furthermore, the hydrogen bonding index (HBI) increase correlates with this stability; the dense interfacial network restricts the segmental mobility of the EVA chains near the filler, thereby counteracting the early decomposition usually triggered by nanocellulose. These findings provide quantitative evidence that the masterbatch approach successfully bridges the thermal stability gap between nanocellulose and thermoplastic matrices.

#### 3.3.3. Crystalline Behavior and Thermal Transitions via DSC Analysis

**Figure 7:**
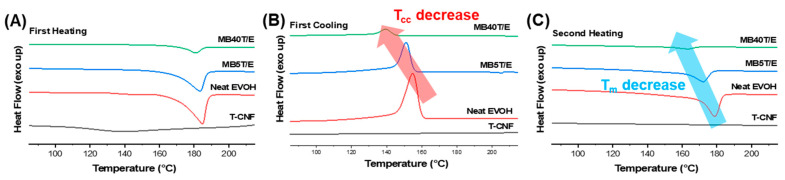
DSC thermograms of neat EVOH and T-CNF/EVOH masterbatches: (**A**) first heating cycle, (**B**) first cooling cycle, (**C**) second heating cycle.

To evaluate the influence of T-CNF incorporation on the crystalline structure and thermal transitions of EVOH, DSC measurements were performed (Figure 7). Thermal transition data from the second heating cycle, including crystallization temperature (*T*_cc_), melting temperature (*T*_m_), and melting enthalpy (Δ*H*_m_), are summarized in Table 5.

Neat EVOH exhibited typical semicrystalline behavior with a sharp melting peak at 183 °C and a melting enthalpy (Δ*H*_m_) of 29.03 Jg^−1^. Upon incorporation of T-CNF, a significant and progressive reduction in both transition temperatures and enthalpy values was observed. Specifically, MB5T/E showed a decrease in Δ*H*_m_ to 9.13 Jg^−1^, while MB40T/E exhibited a near-complete suppression of crystalline domain formation with a Δ*H*_m_ of only 0.52 Jg^−1^.

These results quantitatively demonstrate that T-CNF exerts a profound disruptive influence on the crystallization process of EVOH. While nanocellulose can act as a nucleating agent in certain low-crystallinity matrices, its role here is reversed due to the high density of interfacial hydrogen bonds [55]. The hydroxyl-rich surfaces of T-CNF fibers form a robust network with EVOH, imposing severe conformational constraints on the polymer chains. This interfacial pinning effect effectively restricts the segmental mobility required for lamellar folding and ordering [56]. At higher T-CNF contents (40 wt%), the dense fibrous network significantly restricts the mobility of EVOH chains, thereby limiting long-range chain rearrangement and suppressing crystal growth.

Interestingly, the observed reduction in crystallinity correlates directly with the enhanced thermal stability confirmed via TGA. To quantitatively assess this structural change, the degree of crystallinity (*X_c_*) was calculated using the following equation:

(1) $$X_{c}\left(\%\right)=\frac{∆\;H_{m}}{∆\;H_{m}{}^{\circ}\;\cdot (1-\;\omega )}\times 100$$

where Δ*H_m_* is the measured melting enthalpy from the DSC second heating cycle, Δ*H_m_*° is the theoretical melting enthalpy of 100% crystalline EVOH (taken as 169.2 J g^−1^ based on the vinyl alcohol, as commonly reported), and ω is the weight fraction of the T-CNF filler.

Based on this calculation, the *X_c_* of neat EVOH (17.2%) decreased significantly to 5.7% for MB5T/E and approached a near-zero amorphous state for MB40T/E. This dramatic transition indicates that the T-CNF/EVOH network facilitates a structural reconfiguration from a crystalline-dominant phase to a more amorphous, yet structurally reinforced, flexible network.

While the loss of crystalline domains might typically suggest a decrease in properties, in this system, the high density of interfacial hydrogen bonds acts as a pseudo-crosslinking site. This leads to a more robust amorphous phase where the restricted segmental mobility of the EVOH chains compensates for the absence of lamellar crystals. Such a transition to an amorphous morphology is highly advantageous for specific industrial applications, as it can simultaneously enhance toughness, optical transparency, and interfacial compatibility within the subsequent EVA matrix.

### 3.4. Mechanical Properties of Melt-Compounded EVA Composites Reinforced with T-CNF/EVOH Masterbatches

EVA composites prepared using T-CNF/EVOH masterbatches exhibited marked improvements in mechanical properties, particularly tensile strength and Young’s modulus, compared to neat EVA. All composites were fabricated by melt blending EVA with T-CNF/EVOH masterbatches at a fixed ratio of 99:1, while the T-CNF content within the masterbatch varied (5, 10, 20, and 40 wt%).

**Figure 8:**
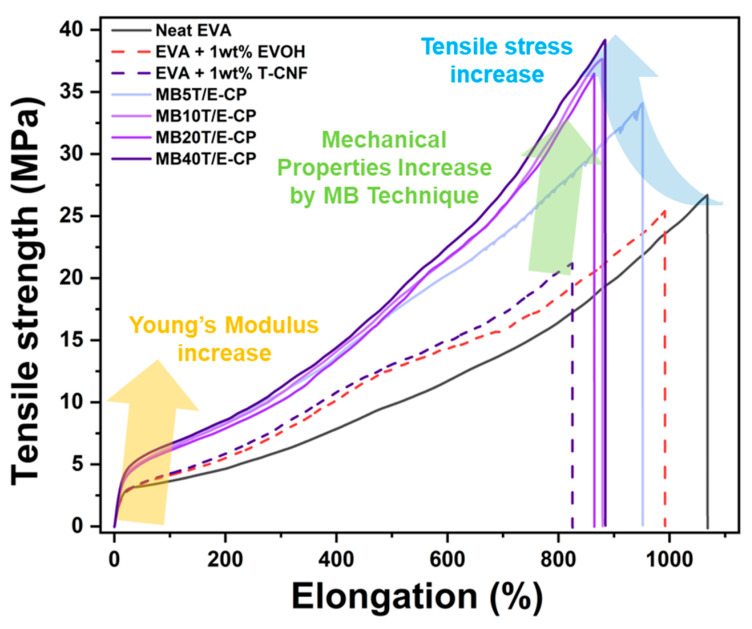
Mechanical properties of neat EVA and composites reinforced with T-CNF/EVOH masterbatches.

First, to validate the effectiveness of the masterbatch strategy, comparative experiments were conducted using direct-blended systems (EVA/T-CNF 99:1 and EVA/EVOH 99:1). As shown in the results (Figure 8), the direct incorporation of either T-CNF or EVOH into the EVA matrix led to a decline in overall mechanical performance compared to Neat EVA.

Specifically, when T-CNF was added directly, both the tensile strength and elongation at break decreased significantly. This is attributed to the poor compatibility between the hydrophilic fillers and the hydrophobic EVA matrix, causing severe agglomeration where clusters act as stress concentrators [57,58,59]. Similarly, the direct addition of 1 wt% EVOH resulted in a reduction in tensile strength to 25 MPa (0.95-fold decrease compared to neat EVA). While the Young’s modulus showed a marginal increase to 27 MPa (1.04-fold increase compared to neat EVA), the improvement was negligible and did not contribute to the reinforcement of the composite. These results highlight that a simple direct-blending approach is insufficient for achieving effective filler dispersion.

In contrast, the masterbatch approach—designed to encapsulate T-CNF within an EVOH shell—enabled significantly higher reinforcement while maintaining structural integrity. Incorporation of MB5T/E-CP yielded substantial enhancements, achieving a tensile strength of 34 MPa and a Young’s modulus of 37 MPa, representing increases of 1.31-fold and 1.42-fold over neat EVA, respectively. Notably, MB5T/E-CP exhibited the highest modulus among all composites, confirming that optimized dispersion at low loading is more critical for stiffness than filler quantity alone [60,61].

At higher T-CNF loadings, the highest tensile strength of 40 MPa was recorded for MB40T/E-CP. Although the elongation at break naturally decreased with increasing filler content (Table 6), the EVOH-based masterbatch strategy effectively mitigated the catastrophic embrittlement observed in the direct-blended samples. This synergy is attributed to the high aspect ratio of T-CNFs and the pre-formed hydrogen bonding within the masterbatch, which together facilitate stable stress transfer and suppress localized stress concentrations.

Notably, the transition of EVOH to a near-amorphous state in the MB40T/E formulation—as confirmed by DSC—does not lead to the typical increase in ductility associated with amorphous polymers. Instead, the interfacial pinning effect of the T-CNF network serves as a pseudo-crosslinking mechanism. While the absence of rigid lamellar crystals theoretically allows for greater chain mobility, the extraordinarily high density of hydrogen bonds between T-CNF and EVOH restricts this movement even more severely than crystalline domains would.

Furthermore, at 40 wt% loading, the densely packed T-CNF network imposes strong spatial confinement on the EVOH chains, significantly restricting their segmental mobility. This confined environment limits chain disentanglement and deformation under applied stress, leading to a mechanically reinforced interfacial region. As a result, the amorphous yet structurally stabilized interface contributes to a pronounced increase in Young’s modulus and tensile strength, indicating that the reinforcement arising from the hydrogen-bonded network effectively dominates over the intrinsic flexibility of the amorphous phase.

The mechanical reinforcement is further supported by the fracture morphology observed in Supplementary Figure S1. While the directly incorporated T-CNF (without encapsulation) led to premature structural failure characterized by severe stress concentration and irregular tearing, the masterbatch-based composites exhibited extensive crack bridging. In these samples, nanofibers effectively linked fractured regions and delayed crack propagation, enabling significant energy dissipation during fracture.

The EVOH-wrapped T-CNF domains allowed for controlled fiber pull-out rather than brittle snapping. This mechanism ensures that the significant enhancement in tensile strength is achieved without a catastrophic loss of structural integrity [36,62,63]. Consequently, the masterbatch approach demonstrates that the EVOH encapsulation layer acts as an essential interfacial bridge, providing a level of structural reinforcement and toughness that raw components cannot achieve through individual direct blending.

## 4. Discussion

This study demonstrated a sustainable and scalable strategy to reinforce EVA composites by physically modifying T-CNF through an EVOH-mediated masterbatch process. The primary advantage of this approach lies in its ability to achieve high-performance reinforcement without the environmental burden of chemical surface treatments.

The significant improvements in mechanical properties—namely the 1.5-fold increase in tensile strength and 1.3-fold increase in Young’s modulus—are fundamentally rooted in the molecular-level structural reconfiguration of the EVOH phase. As evidenced by DSC analysis, the high density of interfacial hydrogen bonds between T-CNF and EVOH successfully suppressed the crystallization of EVOH, transitioning it into a reinforced amorphous state.

Contrary to the typical behavior of amorphous polymers, which exhibit increased ductility, the MB40T/E system showed enhanced stiffness. This is attributed to the interfacial pinning effect, where the robust hydrogen-bonding network acts as a pseudo-crosslinking mechanism, restricting segmental mobility even more effectively than crystalline domains. This effect is further reinforced by the confinement of polymer chains within the densely distributed fibrous network, which suppresses chain disentanglement and mitigates localized stress concentrations during deformation.

Furthermore, the TGA results highlight the critical importance of the stoichiometric balance between the filler and the wrapping agent. While high-shear processing successfully dispersed the T-CNFs in the MB40T/E formulation, the resulting starvation of the EVOH layer—where the available polymer was spread too thin to fully encapsulate the vast surface area—led to lower thermal stability compared to the MB5T/E system. This suggests that the thickness and density of the protective interface are the primary determinants of thermal resistance, whereas physical dispersion dictates the overall mechanical integrity.

While the masterbatch strategy facilitates efficient load transfer and crack bridging, as observed in the fracture morphology, the hydrophilic nature of the components remains a challenge. Preliminary hydrothermal assessments indicate a decrease in properties under high-humidity conditions. Therefore, while this versatile approach holds great promise for a wide range of hydrophobic thermoplastic matrices, future research focusing on complementary hydrophobic surface modifications will be essential for demanding industrial applications, such as long-term stable photovoltaic encapsulants.

## 5. Conclusions

This study presents a simple yet powerful masterbatch strategy to reinforce EVA using EVOH-wrapped T-CNFs:

- T-CNFs were pre-encapsulated in EVOH to create a high-concentration masterbatch, preventing fiber aggregation during melt-processing.
- Even at an ultra-low loading of 0.05 wt%, the composites achieved a 1.42-fold increase in Young’s modulus and a 1.54-fold increase in tensile strength.
- The structural similarity between EVA and EVOH ensured strong interfacial adhesion, transforming masterbatch particles into effective reinforcing nodes.
- This physical modification approach offers a scalable and sustainable route for high-performance industrial applications like solar cell encapsulation.

## Figures and Tables

**Table 1:** FTIR peak shifts of hydrogen bonding and molecular interactions in T-CNF/EVOH masterbatches.

	MB5T/E	MB40T/E	EVOH	T-CNF
Vibration Mode	Wavenumber (cm^−1^)			
–OH stretching	3329	3321	3335	3360
C–O–C asymmetric stretching	1136	1137	-	1160
C–O stretching	1090	1087	1090	1108
C–OH stretching and bending	1029	1033	-	1035
HBI ^a^	6.30	8.23	3.01	8.41

^a^ HBI (hydrogen bonding index) was calculated as the ratio of the integrated areas of hydrogen-bonded (A_bonded_, at ~3350 cm^−1^) to free (A_free_, at ~3620 cm^−1^) hydroxyl groups (HBI = A_bonded_/A_free_).

**Table 2:** Composition of T-CNF in Masterbatch (MB) and Final Composites.

	T-CNF Loading in MB Used (*w*/*w*%)	T-CNF Loading in MB (wt%)	T-CNF Loading in Composite (wt%)
Neat EVA	-	-	-
MB5T/E-CP ^a^	5	4.76	0.05
MB10T/E-CP	10	9.09	0.091
MB20T/E-CP	20	16.67	0.167
MB40T/E-CP	40	28.57	0.286

^a^ The naming of the EVA composites was derived from the corresponding T-CNF/EVOH masterbatch formulations used for blending.

**Table 3:** *T*_d5_ values of T-CNF and T-CNF/EVOH masterbatches.

	*T*_d5_ (°C)
T-CNF	233
MB5T/E	307 (+74) ^a^
MB40T/E	250 (+17)

^a^ Values in parentheses represent the change in *T*_d5_ compared to neat T-CNF (*T*_d5_ = 233 °C).

**Table 4:** Thermal stability of neat EVA and MB/EVA composites.

	*T*_d3_ (°C)	*T*_d5_ (°C)	*T*_d10_ (°C)	*T*_max_ (°C)
Neat EVA	381	397	450	510
MB5T/E-CP	379 (−2) ^a^	395 (−2)	449 (−1)	509 (−1)
MB40T/E-CP	377 (−4)	391 (−6)	447 (−3)	503 (−7)

^a^ Values in parentheses represent the change in *T*_d_ relative to neat EVA.

**Table 5:** Thermal transition temperatures of EVOH and T-CNF/EVOH masterbatches.

	*T*_cc_ (°C)	*T*_m_ (°C)	Δ*H*_m_ (J g^−1^)
Neat EVOH	158	183	29.03
MB5T/E	151 (−7) ^a^	176 (−7)	9.13
MB40T/E	141 (−17)	171 (−12)	0.52

^a^ Values in parentheses represent the change in temperatures relative to neat EVOH.

**Table 6:** Tensile strength and Young’s modulus of EVA composites reinforced with T-CNF/EVOH masterbatches.

	Tensile Strength at Break (MPa)	Young’s Modulus (MPa)
Neat EVA	26	26
EVA + EVOH	25 (0.95-fold) ^a^	27 (1.04-fold)
EVA + T-CNF	21 (0.81-fold)	30 (1.15-fold)
MB5T/E-CP	34 (1.31-fold)	37 (1.42-fold)
MB10T/E-CP	37 (1.42-fold)	33 (1.26-fold)
MB20T/E-CP	37 (1.42-fold)	32 (1.23-fold)
MB40T/E-CP	40 (1.54-fold)	34 (1.31-fold)

^a^ Values in parentheses indicate the increase relative to neat EVA.

## Data Availability

The original contributions presented in this study are included in the article. Further inquiries can be directed to the corresponding author.

## Supplementary Materials

The following supporting information can be downloaded at: https://www.mdpi.com/article/10.3390/polym18020167/s1, Figure S1: SEM images of fracture surface of (A) neat EVA, (B) EVA + 1 wt% T-CNF, (C) MB5T/E-CP, and (D) MB40T/E-CP after UTM testing.
