# Supplementary material for: Mechanical Reinforcement of Ethylene Vinyl Acetate (EVA) Nanocomposites Prepared from Masterbatch of Cellulose Nanofibers Wrapped with Ethylene Vinyl Alcohol (EVOH)
Source: Polymers (Basel). 2026 Jan 8;18(2):167. doi: 10.3390/polym18020167 (PMC12846184; doi:10.3390/polym18020167)
Supplement: Supplementary file 1 [file polymers-18-00167-s001.zip › polymers-4013314-supplementary.pdf]

Supplementary Materials: Mechanical reinforcement of ethylene vinyl acetate (EVA) nanocomposites prepared from masterbatch of cellulose nanofibers wrapped with ethylene vinyl alcohol (EVOH)

Hyungrai Kim [1], Hyewon Lee [1], Seokkyoo Seo [2], Heejung Jang [2] and Jeyoung Park [1,*]

Characterization of Fracture Morphology

To investigate the reinforcement effect and interfacial bonding, the cross-sectional fracture surfaces of the fractured dog-bone nanocomposite specimens were observed via SEM. After performing the tensile test until failure, the fractured ends were collected, and their cross-sections were platinum-coated for high-resolution SEM imaging.

While Neat EVA (Figure S1A) exhibits a typical smooth and featureless fracture surface, the masterbatch-reinforced composites show a significantly more complex morphology. In MB5T/E-CP (Figure S1C) and MB40T/E-CP (Figure S1D), the EVOH-encapsulated T-CNF particles are observed as distinct nodes firmly anchored within the EVA matrix. Even after the high-strain deformation typical of dog-bone specimens during UTM testing, these particles remain well-embedded without debonding. The structural similarity between EVA and the EVOH wrapping layer facilitates robust interfacial adhesion. This allows the masterbatch particles to act as stress-concentrating nodes that effectively bridge micro-cracks and impede crack propagation, correlating with the observed 1.54-fold increase in tensile strength. By the way, in the direct blend (Figure S1B), the absence of the EVOH mediator leads to poor fiber-to-matrix adhesion, evidenced by fiber pull-out and interfacial voids on the fracture surface.

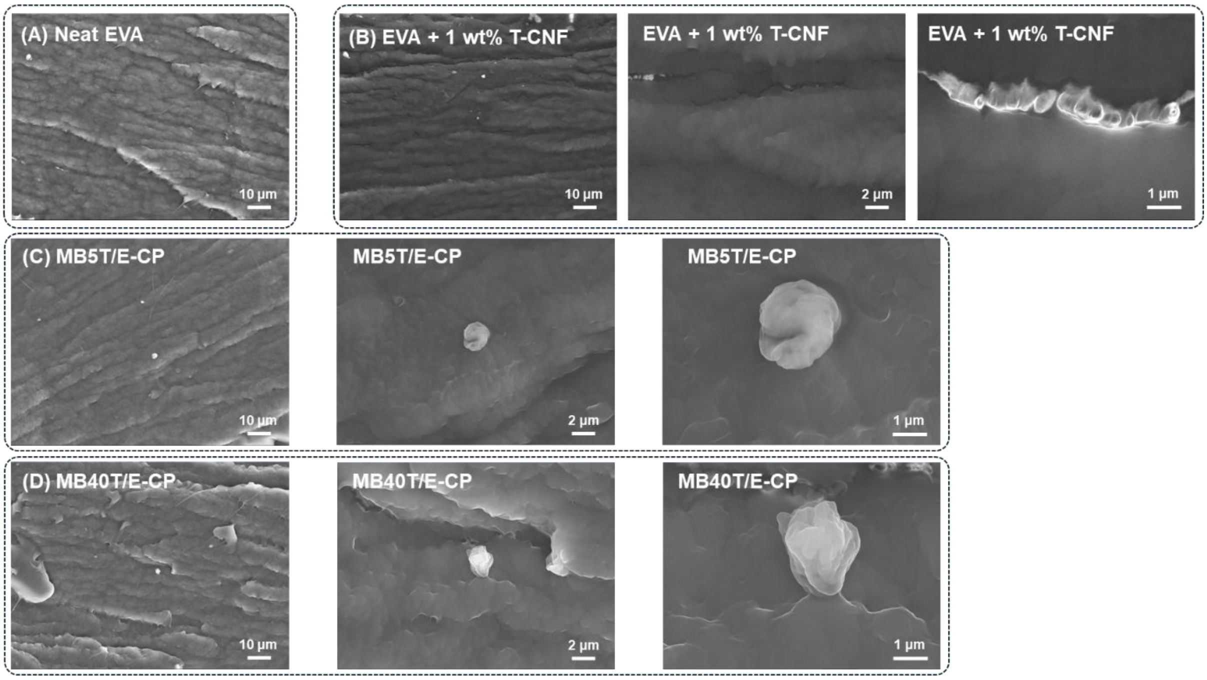

Figure S1. SEM images of fracture surface of (A) neat EVA, (B) EVA + 1 wt% T-CNF, (C) MB5T/E-CP, and (D) MB40T/E-CP after UTM testing
